# Sustainable Exopolysaccharide Production by *Rhizobium viscosum* CECT908 Using Corn Steep Liquor and Sugarcane Molasses as Sole Substrates

**DOI:** 10.3390/polym15010020

**Published:** 2022-12-21

**Authors:** Eduardo J. Gudiña, Márcia R. Couto, Soraia P. Silva, Elisabete Coelho, Manuel A. Coimbra, José A. Teixeira, Lígia R. Rodrigues

**Affiliations:** 1 CEB-Centre of Biological Engineering, University of Minho, 4710-057 Braga, Portugal; 2 LABBELS-Associate Laboratory, Braga/Guimarães, Portugal; 3 LAQV-REQUIMTE, Department of Chemistry, University of Aveiro, 3810-193 Aveiro, Portugal

**Keywords:** Enhanced Oil Recovery, polymer flooding, xanthan gum, heavy oil, pseudoplastic fluid, Herschel–Bulkley model, circular economy, agro-industrial residues

## Abstract

Microbial exopolysaccharides (EPS) are promising alternatives to synthetic polymers in a variety of applications. Their high production costs, however, limit their use despite their outstanding properties. The use of low-cost substrates such as agro-industrial wastes in their production, can help to boost their market competitiveness. In this work, an alternative low-cost culture medium (CSLM) was developed for EPS production by *Rhizobium viscosum* CECT908, containing sugarcane molasses (60 g/L) and corn steep liquor (10 mL/L) as sole ingredients. This medium allowed the production of 6.1 ± 0.2 g EPS/L, twice the amount produced in the standard medium (Syn), whose main ingredients were glucose and yeast extract. This is the first report of EPS production by *R. viscosum* using agro-industrial residues as sole substrates. EPS_CSLM_ and EPS_Syn_ exhibited a similar carbohydrate composition, mainly 4-linked galactose, glucose and mannuronic acid. Although both EPS showed a good fit to the Herschel–Bulkley model, EPS_CSLM_ displayed a higher yield stress and flow consistency index when compared with EPS_Syn_, due to its higher apparent viscosity. EPS_CSLM_ demonstrated its potential use in Microbial Enhanced Oil Recovery by enabling the recovery of nearly 50% of the trapped oil in sand-pack column experiments using a heavy crude oil.

## 1. Introduction

Microbial exopolysaccharides (EPS) have a wide range of potential uses in the food, pharmaceutical, cosmetics and petroleum industries, among others, where they can act as thickening, film-forming, gelling, emulsion-stabilizing, flocculating, texture-improving or mobility control agents [1,2,3,4,5,6]. Although synthetic polymers and biopolymers derived from plants dominate the global market, microbial EPS (e.g., xanthan gum, gellan gum, levan, dextran, scleroglucan, curdlan, pullulan) have garnered increasing interest in the recent years [3,7,8]. For example, the global xanthan gum market is expected to grow from 1000 million USD in 2019 to 1500 million USD in 2027 [9].

Microbial EPS, which can be produced from renewable resources, exhibit less environmental impact when compared with synthetic polymers, and their production is faster than those obtained from plants. Furthermore, they usually display excellent rheological properties, stability at a wide range of environmental conditions, and a broad variety of chemical structures, which provides them different physicochemical properties [3,6,10,11,12,13,14]. Microbial EPS usually exhibit remarkable biological activities (e.g., antitumor, antimicrobial, antioxidant, anti-inflammatory, immunomodulatory or prebiotic activity) that are not present in synthetic polymers [1,7,9,15]. Therefore, some of them could be incorporated in high-value products such as pharmaceuticals, cosmetics, medical devices or functional foods [1,3,8,16,17].

Despite all these advantages, the significant costs involved in producing microbial EPS limit their use. In the last few years, the increasing demand for biopolymers has prompted the search for new molecules with enhanced rheological properties, as well as the development of more efficient and cost-effective manufacturing procedures, in order to make them more appealing to the industry [1,4,18,19,20]. The price of the culture medium has a considerable impact on the overall cost of microbial EPS production. Therefore, the use of alternative low-cost substrates is crucial to reduce the price of the final product. Accordingly, several wastes and by-products generated by agro-industrial processes have been assessed as inexpensive substrates for EPS production by various microorganisms [4,8,12,13,15,16,17,19,21,22].

Among their applications, polymers are widely used by the oil industry to increase the recovery of crude oil from mature reservoirs. After primary and secondary oil recovery operations, usually less than 50% of the original oil in place is recovered from the oil fields [2,23]. In order to mobilize the remaining oil, the application of tertiary oil recovery techniques (i.e., Enhanced Oil Recovery (EOR)) is necessary, which entails injecting chemical compounds (usually polymers, surfactants, alkalis or mixtures of them) into the oil wells [2,6,24]. Among the different EOR approaches, polymer flooding is one of the most favorable. Polymers increase the viscosity of water injected to recover the entrapped oil, which reduces the difference in mobility between water and crude oil, improving the efficiency of oil displacement during water flooding [24,25]. Synthetic polymers (e.g., partially hydrolyzed polyacrylamide (HPAM)) are widely used by the petroleum industry for polymer flooding. However, in recent years, interest in the utilization of microbial EPS (mainly xanthan gum) has increased, as part of a technology known as Microbial Enhanced Oil Recovery (MEOR), where microorganisms and their metabolites are used instead of the chemical compounds used in CEOR [5,11,20,23].

The EPS produced by *Rhizobium viscosum* CECT908 (formerly *Arthrobacter viscosus*) has been scarcely studied since its chemical characterization in the 1960s [26,27,28]. There are few reports regarding its application in the removal of organic compounds and heavy metals from wastewater [29], and there are no previous studies regarding the use of low-cost substrates to improve its productivity or reduce the production costs. Only the effect of the operational conditions (pH, temperature, agitation rate) and the use of different carbon sources (glucose, xylose) at different concentrations on EPS production have been studied [29,30,31].

In our previous work, *R. viscosum* EPS exhibited a huge potential for application in MEOR [2]. In an effort to reduce the production costs, agro-industrial by-products were used to formulate a low-cost culture medium for its production. The rheological properties and the structure of the EPS produced in the conventional and the low-cost medium were studied, and the applicability of the EPS produced in the low-cost medium in oil recovery was assayed in sand-pack columns.

## 2. Materials and Methods

### 2.1. Exopolysaccharide Production

#### 2.1.1. Microorganism, Culture Medium and Culture Conditions

The EPS-producing strain *R. viscosum* CECT908 was stored at −80 °C in a synthetic medium supplemented with glycerol (15%, *v*/*v*). The composition of the synthetic medium was as follows (g/L): glucose, 25; yeast extract, 3; K_2_HPO_4_, 2; MgSO_4_.7H_2_O, 0.1; and pH 7 [30]. Erlenmeyer flasks (500 mL) containing 200 mL of synthetic medium were incubated at 28 °C and 180 rpm for 168 h after inoculation with 1% (*v*/*v*) of a pre-culture prepared from frozen stocks (grown for 24 h under the same conditions). In order to evaluate EPS production, bacterial growth and sugars consumption, samples were taken every 24 h. The apparent viscosity of the samples was measured as described in the following sections. In some cases, the EPS produced was recovered and quantified, as described below. Bacterial growth was determined by the plate count technique. Serial dilutions of culture samples were performed in a saline buffer (9 g NaCl/L) supplemented with Tween 80 (0.1 g/L) and plated in MA medium, with the following composition (g/L): glucose, 20; peptone, 5; malt extract, 3; yeast extract, 3; agar, 20; and pH 7. The plates were incubated at 28 °C and the number of cells was expressed as Log_10_ colony-forming units (CFU) per mL. Sugars concentration was determined through HPLC, as described below. All experiments were performed at least in triplicate.

#### 2.1.2. Alternative Culture Media

Sugarcane molasses and corn steep liquor (CSL), provided by local industries, were evaluated as low-cost substrates for EPS production. Different culture media were prepared containing different concentrations of both substrates. All media were adjusted to pH 7 using 6 M HCl or 10 M NaOH, and cultures were performed as described in the previous section. EPS production, bacterial growth and sugar consumption were evaluated as described for the synthetic medium. All assays were performed in triplicate.

#### 2.1.3. Recovery and Purification of Exopolysaccharides

The culture broth samples were centrifuged (9400× *g*, 30 min) for cell separation. Whenever required, viscous samples were diluted with demineralized water (between three and five times) in order to reduce their viscosity before centrifugation and allow the sedimentation of the cells. The crude EPS was precipitated through the addition of hexadecyltrimethylammonium bromide (CTAB) to the cell-free supernatants, and subsequently purified as described by Novak and co-workers [31]. The purified EPS was freeze-dried, weighed, and stored at −20 °C for further studies.

#### 2.1.4. Analytical Techniques

A Discovery HR1 rheometer (TA Instruments, New Castle, DE, USA), provided with a cone-plate geometry (diameter 60 mm; angle 2.006°; gap 0.064 mm), was used to measure the apparent viscosity (*η*, mPa s) and shear stress (*τ*, mPa) of culture broth samples, EPS and xanthan gum (Sigma-Aldrich Co., Saint Louis, MO, USA) solutions at different shear rates ($\dot{\gamma }$, 0.1–300 s^−1^). The measurements were performed at 25 °C, and the viscosity values presented correspond to a shear rate of 1.4 s^−1^, unless indicated otherwise. Each sample was analyzed in triplicate.

The concentration of sugars and lactic acid in the cell-free supernatants was determined using an HPLC system equipped with an Aminex^®^ HPX-87H (300 × 7.8 mm, Bio-Rad, Des Plaines, IL, USA) column coupled to an UV detector (K-2501, Knauer, Highwood, IL, USA) and a refractive index detector (RI-2031 Plus, JASCO, Oklahoma City, OK, USA). H_2_SO_4_ (5 mM) was used as mobile phase (flow rate 0.6 mL/min), and the column temperature was set at 35 °C. Calibration curves prepared using pure compounds were used to calculate the concentration of different sugars and lactic acid. Chromatograms were analyzed using the software Varian Star 6 Chromatography Workstation (Varian, Palo Alto, CA, USA).

### 2.2. Exopolysaccharides Characterization

#### 2.2.1. Sugars Analysis

Neutral sugars were released from EPS samples by treatment with 72% (*w*/*w*) H_2_SO_4_ (3 h, room temperature), followed by hydrolysis (2 M H_2_SO_4_, 1 h, 120 °C) using a heating block. The sugars were then derivatized to their alditol acetates and analyzed using a gas chromatograph equipped with a flame ionization detector (GC–FID PerkinElmer-Clarus 400) and provided with a DB-225 column [32,33]. Samples were analyzed in duplicate.

Uronic acids (UA) were determined using a colorimetric method according to a modification [34] of the method proposed by Blumenkrantz and Asboe-Hansen [35]. Samples were prepared by pre-hydrolysis (72% (*w*/*w*) H_2_SO_4_, 3 h, room temperature) followed by hydrolysis (1 M H_2_SO_4_, 1 h, 100 °C) using a heating block. A calibration curve was prepared using d-galacturonic acid as standard. Samples were analyzed in triplicate.

#### 2.2.2. Linkage Analysis of Carbohydrates

Methylation analysis was used to identify glycosidic linkages following a protocol described by Reis and co-workers [36]. Briefly, EPS were methylated using CH_3_I, dissolved in CHCl_3_:MeOH (1:1, *v*/*v*), and dialyzed against 50% EtOH. Subsequently, methylated EPS were carboxyl-reduced in order to identify the uronic acid derivatives. The obtained product was completely dried and reduced with LiAlD_4_ (lithium aluminum deuteride) in dried tetrahydrofuran at 65 °C for 4 h. EtOH and water were used to destroy the remaining reagent, the pH was adjusted to neutral value (2 M H_3_PO_4_), and further extraction was performed using CHCl_3_:MeOH (2:1, *v*:*v*). After solvent evaporation, the product obtained was hydrolyzed (trifluoroacetic acid 3 M), reduced (NaBD_4_ (sodium borodeuteride)) and acetylated. The partially methylated alditol acetates (PMAAs) obtained were analyzed by gas chromatography–mass spectrometry (GC–qMS) according to Reis and co-workers [36]. For that purpose, a Shimadzu GCMS-QP2010 Ultra (Shimadzu Co., Tokyo, Japan), equipped with a HT5 (Trajan Scientific, Victoria, Australia) capillary column (30 cm length × 0.25 mm i.d., 0.1 μm film thickness), was used. Samples were dissolved in 70 μL of anhydrous acetone, and injection was performed in split mode (split ratio 2.0) with the injector operating at 250 °C. The initial temperature was set at 80 °C, and a linear increase (7.5 °C/min up to 140 °C) was applied. After standing for 5 min at 140 °C, a linear temperature increase (0.2 °C/min up to 143.2 °C) was applied, followed by a linear increase up to 250 °C (15 °C/min), standing 5 min at this temperature. Helium was used as a carrier gas (flow rate 1.84 mL/min), with a column head pressure of 124.1 kPa. The GC was connected to an Agilent 5973 mass quadrupole selective detector, operating with an electron impact mode at 70 eV, and scanning the range *m*/*z* 50–700 in a 1 s cycle in a full scan mode acquisition.

#### 2.2.3. Acetylation Degree

Methyl and acetyl groups were quantified as methanol and acetic acid, respectively, as described by Nunes et al. [37]. Briefly, EPS samples (1 mg) were dissolved in water (250 µL) and sonicated (10 min) in a water bath at room temperature. 1-propanol (50 µL, 0.2%) was added to the samples as internal standard. Subsequently, 100 µL of 2 M NaOH were added to the samples and saponification was promoted by incubation at 25 °C for 1 h. The reaction was stopped by the addition of 150 µL of 2 M HCl, and the solution was filtered (0.2 µm nylon filter). Finally, the filtrate was injected in a Hewlett-Packard 5890 series II GC-FID, equipped with a DB-Wax column (J&W) (30 cm length × 0.53 mm i.d. × 1.0 mm film thickness). Hydrogen was used as the carrier gas (flow rate 6 mL/min). Calibration curves were prepared using pure compounds at concentrations between 100 and 500 mg/L. Estimated concentrations were determined by comparing the obtained peak areas with the area of the known concentration of the internal standard. Samples were prepared in duplicate, and each run was performed twice.

### 2.3. Oil Recovery Assays

Sand-pack columns were used to evaluate the performance of the EPS produced by *R. viscosum* CECT908 (grown in the optimized alternative culture medium) in MEOR. The columns, the sand and the methodology used were the same as the ones described in our previous work [2]. The viscosity of the crude oil used (*η*_40 °C_ = 496 ± 54 mPa s) was determined using a hybrid rheometer (Discovery HR1, TA Instruments, USA). Control assays were performed by injecting demineralized water or xanthan gum (Sigma-Aldrich Co., USA) solutions, instead of EPS. All assays were performed at 40 °C in triplicate.

## 3. Results and Discussion

### 3.1. Exopolysaccharide Production by Rhizobium viscosum CECT908 Grown in Synthetic Medium

**Figure 1:**
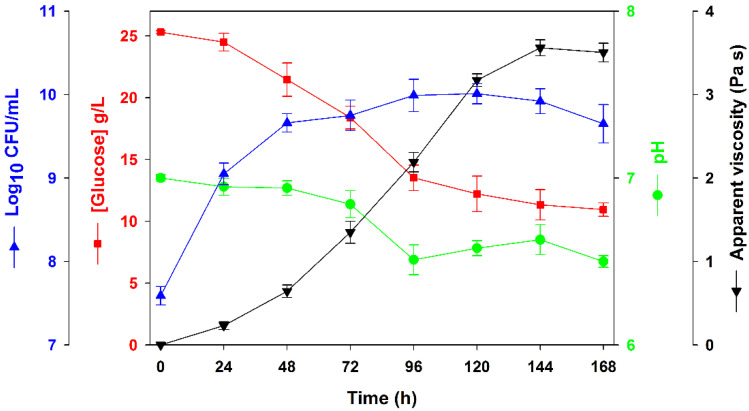
Time course of apparent viscosity (Pa s), colony-forming units (Log_10_ CFU/mL), glucose concentration (g/L) and pH in assays performed with *Rhizobium viscosum* CECT908 grown in synthetic medium in flasks at 28 °C and 180 rpm. The results represent the average of triplicate experiments ± standard deviation.

When *R. viscosum* was grown in the synthetic medium, the highest apparent viscosity (3560 ± 96 mPa s) was achieved after 144 h (Figure 1), corresponding to the production of 3.0 ± 0.2 g EPS/L. The number of cells increased fast in the first 48 h, and after that remained almost stationary, achieving 8.3 × 10^9^–1.0 × 10^10^ CFU/mL between 96 and 144 h. A slight decrease was observed in the pH of the culture medium along the fermentation, dropping from 7.0 to values around 6.5. After 144 h of growth, 11.3 ± 1.2 g/L of residual glucose remained in the culture medium, corresponding to a glucose consumption rate of 54.8%. However, reducing the initial concentration of glucose from 25 to 20 g/L resulted in lower apparent viscosity values (2595 ± 81 mPa s) and a lower EPS production (2.2 ± 0.3 g/L). This is in agreement with previous studies performed with *R. viscosum* CECT908, where the highest EPS production was achieved using 25 or 30 g/L of carbon source (glucose or xylose) [30,31]. The presence of a high concentration of residual sugar in the culture medium is in accordance with previous reports [16,18,38,39]. Contrarily, other EPS-producing strains exhibit sugar consumption rates higher than 90% [8,17,19,22,24,25].

### 3.2. Sugarcane Molasses as an Alternative Carbon Source for Exopolysaccharide Production

To reduce the production costs of the *R. viscosum* EPS, glucose was replaced by sugarcane molasses in the synthetic medium. Sugarcane molasses, which is commonly used as a carbon source in different biotechnological processes, is a residue from sugar refineries, whose exploitation is economically unfavorable [40]. The molasses used in this work contained sucrose (435 ± 8 g/kg), glucose (119 ± 5 g/kg) and fructose (51 ± 2 g/kg). Besides carbohydrates, sugarcane molasses contains metal ions (calcium, cooper, iron, magnesium, manganese, phosphorous, potassium and sodium), vitamins, organic acids and amino acids, which improve bacterial growth [8,22,38,40].

**Figure 2:**
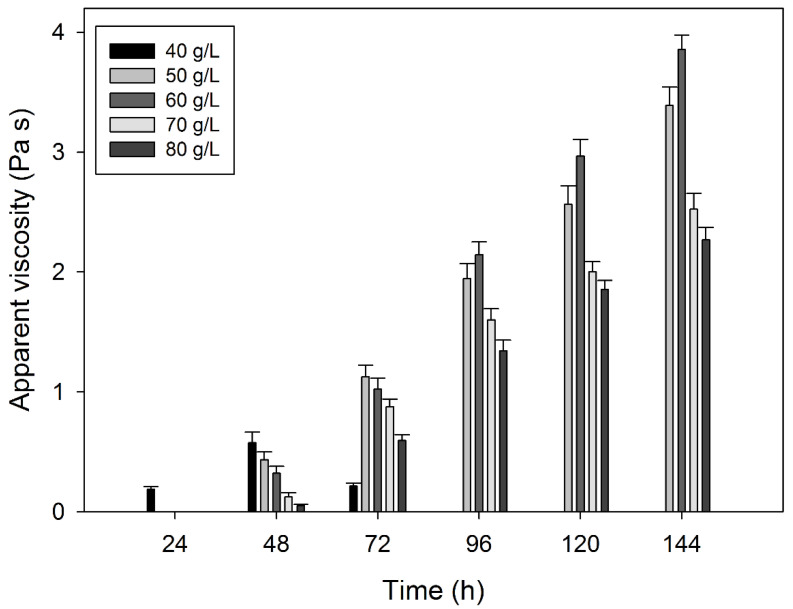
Apparent viscosity values (Pa s) obtained in cultures of *Rhizobium viscosum* CECT908 grown in synthetic medium containing different concentrations of sugarcane molasses (40–80 g/L) instead glucose. The assays were performed at 28 °C and 180 rpm. The results represent the average ± standard deviation of three independent experiments.

Different culture media were prepared containing different concentrations of molasses (40–80 g/L). The cultures were performed as described for the synthetic medium, and EPS production was determined by measuring the apparent viscosity of the culture medium at different time intervals (Figure 2).

As can be seen in Figure 2, the lowest concentration of molasses tested (40 g/L) resulted in a rapid increase in the apparent viscosity of the culture medium when compared with the other media, achieving its maximum after 48 h of growth (576 ± 87 mPa s). However, after that point, the apparent viscosity decreased. As the concentration of molasses increased, the time necessary to achieve the highest apparent viscosity value also increased, probably due to an adaptation of the microorganism to the substrate (pre-cultures were performed in the synthetic medium, which contains glucose as carbon source, whereas sugarcane molasses contains glucose, fructose and sucrose, with sucrose being the most abundant sugar). With the media containing 50 and 60 g molasses/L, similar apparent viscosity values to those obtained with the synthetic medium were achieved after 144 h of growth (3389 ± 154 mPa s and 3854 ± 122 mPa s, respectively). The media containing 70 and 80 g molasses/L led to worse results, i.e., lower values of apparent viscosity. According to these results, a concentration of 60 g/L of sugarcane molasses (which corresponded to 26.1 ± 0.9 g sucrose/L, 7.2 ± 0.6 g glucose/L and 3.1 ± 0.3 g fructose/L) was selected to perform the following studies.

Molasses from sugar beet and sugarcane were identified as suitable carbon sources for the production of xanthan gum, gellan gum, welan gum, pullulan and pantoan by *Xanthomonas campestris*, *Sphingomonas* spp., *Alcaligenes* spp., *Aureobasidium pullulans* and *Pantoea* sp. [8,15,21,22,38,41]. Other alternative carbon sources used for the production of different EPS are summarized in Table 1. However, some of those substrates required a pretreatment (e.g., acid or enzymatic hydrolysis) to hydrolyze disaccharides or complex carbohydrates into monosaccharides [13,15,18,38]. In other cases, inhibitory compounds (e.g., heavy metals, organic acids, phenolic compounds or furan aldehydes) present in those substrates had to be removed before their use as carbon source [18,38,40]. On the contrary, in our case, sugarcane molasses was used as carbon source without any previous treatment, which is crucial from an economic perspective, in order to lower production costs.

### 3.3. Corn Steep Liquor as an Alternative Nitrogen Source for Exopolysaccharide Production by Rhizobium viscosum CECT908

CSL, the main residue of corn starch production, was evaluated as an alternative nitrogen source for EPS production by *R. viscosum*. CSL is rich in proteins, amino acids, vitamins and trace elements, making it a great nutritional supplement for numerous biotechnological processes and an inexpensive substrate when compared with yeast extract [12,19,42,43]. The CSL used in this work also contained glucose (28 ± 2 g/L) and fructose (29 ± 1 g/L). In the synthetic medium containing 60 g/L of molasses instead of glucose, yeast extract was replaced by different concentrations of CSL (10, 20, 30, 50 and 100 mL/L). As in the previous assays, EPS production was assessed by measuring the apparent viscosity of the culture medium at different time intervals. Regarding the media containing 50 and 100 mL CSL/L, the apparent viscosity did not increase during the fermentation (even after 168 h of growth), indicating that there was no production of EPS. In the media containing 20 and 30 mL CSL/L, the highest apparent viscosity values (6925 ± 128 mPa s and 4675 ± 172 mPa s, respectively) were achieved after 144 h of growth. The best result was obtained using 10 mL CSL/L, leading to a viscosity of the culture medium of 10,531 ± 292 mPa s after 144 h of growth (almost three times the value obtained using the synthetic medium).

**Figure 3:**
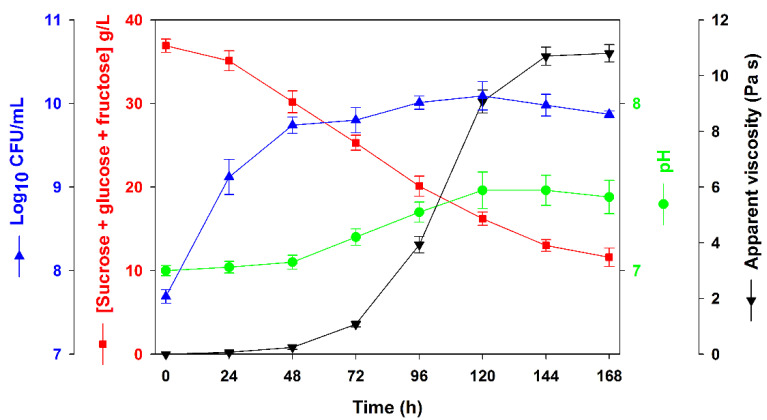
Time course of apparent viscosity (Pa s), colony-forming units (Log_10_ CFU/mL), sugars consumption (g/L) and pH in assays performed with *Rhizobium viscosum* CECT908 grown in the alternative low-cost medium (CSLM) at 28 °C and 180 rpm. The results represent the average of triplicate experiments ± standard deviation.

Finally, a simplified low-cost culture medium (CSLM), containing CSL (10 mL/L) and sugarcane molasses (60 g/L) as sole ingredients, was evaluated. In this case, the apparent viscosity values obtained after 144 h of growth were similar to those achieved with the same culture medium supplemented with K_2_HPO_4_ and MgSO_4_ (10,697 ± 326 mPa s (Figure 3)), and the amount of EPS produced was 6.1 ± 0.2 g/L. Despite the higher EPS production achieved, the bacterial growth profile and CFU/mL obtained using this medium were similar to those observed in the synthetic medium (Figure 1 and Figure 3). Regarding the pH, it exhibited an opposite tendency when compared with the synthetic medium, with a slight increase along the bacterial growth. After 144 h, 13.0 ± 0.7 g/L of residual sucrose remained in the culture medium, whereas the concentration of glucose and fructose was almost zero (total sugars consumption rate 64.7%). As can be seen in Table 1, EPS yields were almost the same in the synthetic and the low-cost (CSLM) medium.

K_2_HPO_4_ and MgSO_4_ are crucial for the proper production of EPS by *R. viscosum*. This was demonstrated by growing *R. viscosum* in a synthetic medium without these salts. When MgSO_4_ was removed from the synthetic medium, no significant increase in the apparent viscosity of the culture medium was observed, indicating that there was no production of EPS; the bacterial growth was lower, and the pH exhibited a similar profile when compared with the synthetic medium (data not shown). In the synthetic medium without K_2_HPO_4_, a similar bacterial growth was observed, in comparison to the synthetic medium, and the apparent viscosity achieved 2170 ± 142 mPa s after 144 h of growth; however, the pH dropped to approximately 5 (data not shown). The fact that EPS production in the low-cost medium was not affected by removing K_2_HPO_4_ and MgSO_4_ may be due to the presence of those elements in CSL or sugarcane molasses [38,40,42]. Phosphate is required for the activation of sugars during EPS synthesis, whereas magnesium is a co-factor of enzymes involved in the metabolism and transport of carbohydrates. Furthermore, K_2_HPO_4_ acts as buffering agent [15,17,40].

The applicability of CSL as an alternative nitrogen source for the production of EPS by different microorganisms has been previously reported. CSL was successfully used to replace yeast extract in the culture medium used for the production of gellan gum by *Sphingomonas paucimobilis* [4], and offered the best results among different agro-industrial wastes evaluated as alternative nitrogen sources for the production of pullulan by *A. pullulans* RBF 4A3 [12,19]. Similar to the results herein reported for *R. viscosum* CECT908, concentrations of CSL higher than 1–2% were detrimental to EPS production, which was attributed to its high lactic acid content [12,19,39]. According to the HPLC analysis, the CSL used in this work contained 123 ± 18 g lactic acid/L, which is in agreement with previous reports [43].

EPS titers between 0.29 and 100 g/L have been reported for different microorganisms, with productivities between 0.19 and 100 g/(L × day) [3,4,8,15,16,17,18,19,20,22,25,38,39,44]. Although EPS titers obtained with *R. viscosum* were lower when compared with those achieved with other microorganisms, it exhibited, in most cases, a higher viscosifying activity, as can be seen in Table 1.

**Table 1:** Summary of the results obtained for the production of different extracellular polysaccharides (EPS) using different by-products and wastes.

Microorganism/ EPS	Carbon Source/ Nitrogen Source	Cultivation Mode	*η* (mPa s)	EPS Titer (g/L)	EPS Yield (g/g Carbon Source)	Productivity (g/(L × Day))	Reference
*Alcaligenes* sp. ATCC31555/ Welan gum	Sugarcane molasses */ Beef extract	Bioreactor	3730 ± 40	41.0 ± 1.4	0.70 ± 0.16	8.16 ± 0.24	[38]
*Alcaligenes faecalis*/Curdlan	Orange peels * + sucrose/ (NH_4_)_2_HPO_4_ + CSL	Bioreactor	-	23.2	0.53	7.73	[18]
*Aureobasidium pullulans* AZ-6/ Pullulan	Sugarcane molasses/ Sugarcane molasses	Flask	-	33.6	0.39	3.7	[22]
*Aureobasidium pullulans* AZ-6/ Pullulan	Hazelnut husk hydrolysate/ (NH_4_)_2_SO_4_	Flask	-	74.3	0.68	10.6	[44]
*Aureobasidium pullulans* RBF 4A3/ Pullulan	Glucose/CSL	Bioreactor	-	88.6	0.68	22.15	[19]
*Enterobacter* A47/FucoPol	Cheese whey */ (NH_4_)_2_HPO_4_	Bioreactor (fed-batch)	≈100	6.4	≈0.02	2.00	[16]
*Enterobacter* A47/FucoPol	Tomato paste **/ (NH_4_)_2_HPO_4_	Bioreactor (fed-batch)	≈100	8.7	-	2.92	[17]
*Pantoea* sp. BCCS 001 GH/Pantoan	Sugar beet molasses/ peptone	Flask	≈2000	9.9 ± 0.5	0.33	4.9	[8]
*Pseudomonas stutzeri* XP1/Dextran	Corn starch/NaNO_3_	Flask	2241	16.0	-	3.2	[20]
*Schizophyllum commune* ATCC38548/Schizophyllan	Date syrup/CSL	Flask	-	8.5 ± 0.2	0.29	1.06	[39]
*Sphingomonas paucimobilis*/ Gellan gum	Glucose/CSL * + urea	Flask	6840	14.4	0.43	7.20	[4]
*Sphingomonas sp.* FM01/ Welan gum	Sugarcane molasses */ Beef extract	Flask	3443	37.7	0.63	12.55	[15]
*Xanthomonas campestris* LRELP-1/ Xanthan gum	Kitchen waste */ Kitchen waste *	Bioreactor	≈500	11.7	0.67	2.93	[25]
*Rhizobium viscosum* CECT908	Glucose/Yeast extract	Flask	3560 ± 96	3.0 ± 0.2	0.22 ± 0.05	0.50 ± 0.03	This study
*Rhizobium viscosum* CECT908	Sugarcane molasses/CSL	Flask	10,697 ± 365	6.1 ± 0.2	0.26 ± 0.09	1.01 ± 0.05	This study

*η*: apparent viscosity. CSL: corn steep liquor. *: pretreated. **: out-of-specification.

### 3.4. Rheological Properties

**Figure 4:**
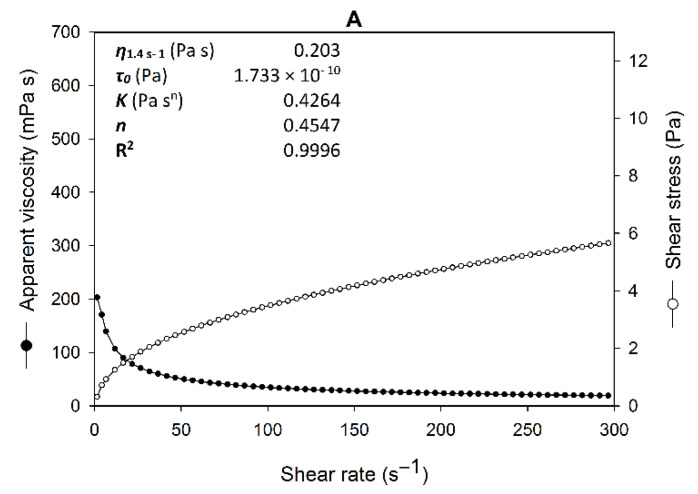

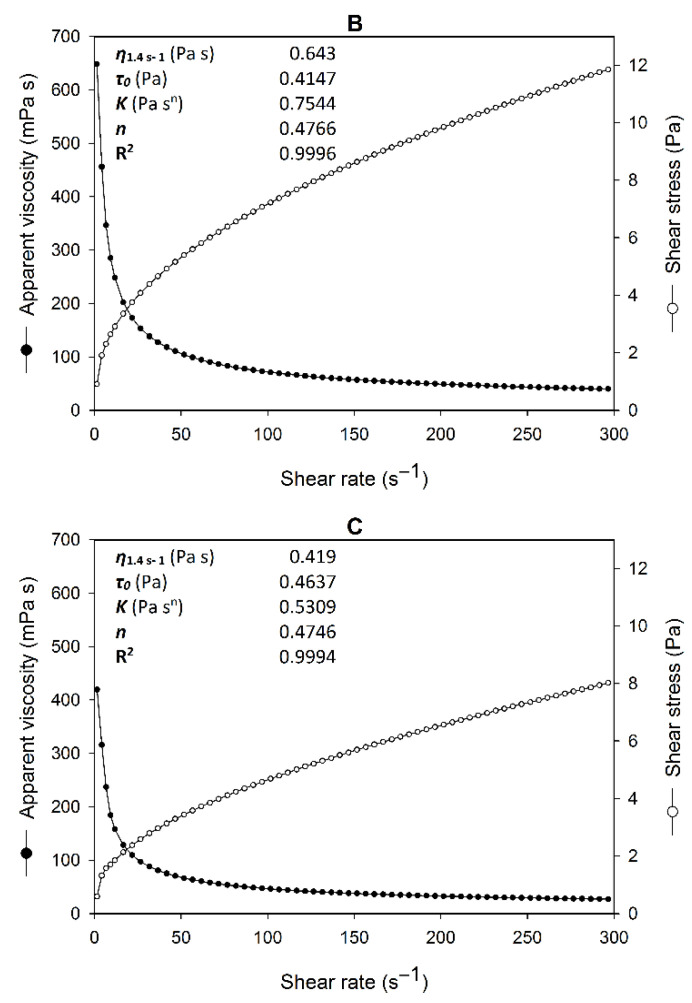
Rheograms of aqueous solutions of the exopolysaccharides produced by *Rhizobium viscosum* CECT908 grown in synthetic medium (EPS_Syn_) (**A**) and alternative low-cost medium (EPS_CSLM_) (**B**), when compared with xanthan gum (**C**) (all of them at a concentration of 1 g/L), and rheological parameters obtained according to the Herschel–Bulkley model. The results represent the average of three independent measurements performed at 25 °C.

The rheograms obtained for aqueous solutions (1 g/L) of EPS_Syn_ (EPS produced in the synthetic medium), EPS_CSLM_ (EPS produced in the alternative low-cost medium) and xanthan gum showed a non-Newtonian pseudoplastic behavior, characterized by a non-linear relationship between shear rate and shear stress, and an inverse relationship between apparent viscosity and shear rate (Figure 4).

Typically, other EPS have been reported to have this behavior. At low shear rates, EPS solutions exhibit high viscosity values because EPS molecules are arranged in intricate and intertwined aggregates. However, as the shear rate moves from low to high values, the apparent viscosity decreases as those aggregates are disorganized and EPS molecules organize themselves along the flow direction [6,8,16,20,24]. The Herschel–Bulkley model describes the relationship between shear stress and shear rate (Equation (1)):

(1) $$\tau =\tau_{0}+K\times {\dot{\gamma }}^{n}$$

where *τ* represents the shear stress (Pa), *τ*_0_ is the yield stress (Pa), $\dot{\gamma }$ is the shear rate (s^−1^), *K* is the flow consistency index (Pa s^n^) and *n* is flow behavior index (dimensionless).

EPS_CSLM_ and EPS_Syn_ exhibited a good fit to the Herschel–Bulkley model (R^2^ = 0.9996), and similar flow behavior indexes (0.4547 and 0.4766, Figure 4A,B). Pseudoplastic fluids are characterized by flow behavior indexes lower than 1. As expected, xanthan gum also showed a pseudoplastic behavior (*n* = 0.4746, Figure 4C). EPS_CSLM_ exhibited a higher flow consistency index when compared with EPS_Syn_, as a result of its higher viscosity, and higher apparent viscosity values than xanthan gum at the same shear rates (Figure 4).

### 3.5. Exopolysaccharides Characterization

Sugars analysis showed a similar carbohydrate composition for EPS_Syn_ and EPS_CSLM_, with higher amounts of galactose, followed by glucose and uronic acids, with the proportion of 1.3:1.0:0.5 (EPS_CSLM_) and 1.3:1.0:0.7 (EPS_Syn_) (Table 2). *R. viscosum* was previously reported to produce an anionic extracellular heteropolysaccharide, whose carbohydrate composition was the same regardless of the carbon source used [26,31]. However, the ratios herein obtained are slightly different from those reported for the EPS produced by *R. viscosum* NRRL B-1973 grown in starch-derived sugars, which possessed an equimolar proportion of these carbohydrates [26]. When *R. viscosum* NRRL B-1973 was grown in glucose, xylose or xylan hydrolysate, similar molar percentages to EPS_CSLM_ and EPS_Syn_ were found, especially when using a xylan hydrolysate as the carbon source (1.2:1.0:0.7) [31]. This polysaccharide was previously reported to contain between 20 and 30% of the dry weight of *O*-acetyl groups, depending on the carbon source used [26,27,28,31]. The quantification of acetic acid (Table 2) showed that the acetyl groups accounted for 18.4% and 21.3% of EPS_CSLM_ and EPS_Syn_ total weight, respectively.

The residues of arabinose and xylose found in EPS_CSLM_ and mannose found in EPS_Syn_ can be derived from the culture media, as CSL has been previously reported to contain arabinoxylans [45], and the synthetic medium contains mannoproteins from a yeast extract [46].

Methylation analysis (Table 3) demonstrated that the EPS produced have a backbone composed of (1→4)-linked-galactosyl residues, which accounts for about 50% of the glycosidic linkages, (1→4)-linked-glucosyl residues (13% for EPS_CSLM_ and 14% for EPS_Syn_) and (1→4)-linked-mannuronosyl residues (11% for EPS_CSLM_ and 17% for EPS_Syn_). This structure is in accordance with the literature, as the EPS produced by *R. viscosum* was previously reported to have a linear structure consisting predominantly of repeating trisaccharide units: *O*-(d-mannopyranosyl uronic acid-(β1→4)-*O*-(d-glucopyranosyl-(β1→4)-d-galactose [28]. The quantified branched residues may result from the acetyl groups that still resisted the alkaline media during methylation analysis, showing acetylation pattern mainly in glucose residues at position *O*-6.

The similar carbohydrate composition of EPS_CSLM_ and EPS_Syn_ (Table 2) does explain their different viscosities (Figure 4A,B). The viscosity of xanthan gum is attributed to its high molecular weight (2–20 × 10^6^ Da), and the rigid and ordered helical conformation of xanthan gum chains in solution [10,21,25]. The high viscosity of EPS_CSLM_ (Figure 4B) can be explained because anionic linear EPS molecules tend to adopt more extended conformations, due to repulsion forces between negative charges, when compared with neutral EPS. Furthermore, high acetylation degrees (Table 2) have been associated with high viscosities [24,31].

### 3.6. Economic Analysis

The use of expensive nutrients (e.g., yeast extract, peptone, glucose) for EPS production results in high production costs. In order to make the production process economically viable, it is necessary to find alternative low-cost carbon and nitrogen sources. The alternative low-cost culture medium (CSLM) developed in this work for EPS production by *R. viscosum* CECT908, contains CSL and sugarcane molasses as the only ingredients, without additional supplements. Furthermore, both substrates were used without the need of any pretreatment, which is beneficial from an economic perspective. Table 4 shows an analysis of the EPS production costs using the synthetic and the low-cost medium. As can be seen, the use of the alternative medium reduced the cost of the nutrients necessary for the production of 1 kg of EPS more than 30 times when compared with the synthetic medium.

### 3.7. Oil Recovery Assays

The applicability of the EPS produced by *R. viscosum* CECT908 in the alternative low-cost medium (EPS_CSLM_) in MEOR was assessed in sand-pack column assays using a heavy crude oil (*η*_40 °C_ = 496 ± 54 mPa s). In order to study the effect of the viscosity of the solution injected in the efficiency of the oil recovery process, aqueous solutions of EPS_CSLM_ and xanthan gum, with apparent viscosities around 50 and 100 mPa s, were prepared. In this case, the apparent viscosity values presented correspond to a shear rate of 9.2 s^−1^, as shear rates between 6 and 10 s^−1^ are commonly used as reference for oil reservoirs [5,6]. The sand-pack columns (280 mL) were first saturated with demineralized water, and after that with crude oil. The amount of oil retained into the columns (OOIP) was between 89 and 96 mL in the different assays, which represents between 95% and 100% of the pore volume of the columns. After the water flooding process (secondary recovery), around 50% of the OOIP was recovered (Table 5). The residual oil entrapped into the columns (between 43 and 50 mL) was the target of the tertiary oil recovery process. EPS_CSLM_ or xanthan gum solutions (200 mL) were injected into the columns, followed by 200 mL of demineralized water. In the control assays, the columns were flooded with 400 mL of demineralized water. As can be seen from the results obtained (Table 5), in the control assays no additional oil was recovered. However, in the assays performed with EPS_CSLM_, almost 50% of the entrapped oil (25–26% of the OOIP) was recovered, regardless of the viscosity of the solution injected. That could be due to the formation of a stable oil displacement front with the lowest EPS_CSLM_ concentration tested (corresponding to an apparent viscosity of 50 mPa s), whose efficiency was not improved by increasing the viscosity of the solution injected up to 100 mPa s. Furthermore, taking into account that around 50% of the entrapped oil was recovered in these assays, in order to recover additional oil, it may be necessary to use of a combination of EPS with surfactants/and or alkalis, in order to reduce the interfacial tension of the oil/sand/water system and change the wettability of the substrate from oil-wet to water-wet [6,20,23,24]. It is important to highlight that EPS_CSLM_ demonstrated a higher efficiency than xanthan gum at the same conditions (Table 5), which is in agreement with previous reports [2].

The shear thinning behavior of EPS_CSLM_ makes it appropriate for use in MEOR. When it is injected into the oil well at high flow rates, it exhibits a low apparent viscosity, which reduces the injection pressure and energy consumption. Inside the oil reservoir, as the flow rate decreases, the apparent viscosity increases again, improving the sweep efficiency [2,5]. EPS_CSLM_ performed well in terms of oil recovery when compared with other EPS. In our previous work, in sand-pack column assays performed using a heavy oil (*η*_40 °C_ = 167 ± 41 mPa s), the EPS produced by *R. viscosum* CECT908 grown in a synthetic medium allowed the recovery of 38% of the entrapped oil, whereas xanthan gum recovered between 30% and 32% [2]. Xu and co-workers [23] studied the applicability of welan gum in MEOR. Using a heavy oil (458 mPa s), tertiary recoveries between 34.5% and 35.7% (corresponding to final recoveries between 71% and 75%) were obtained [23]. The EPS diutan gum produced by *Sphingomonas* sp. allowed tertiary oil recoveries between 20% and 25% (final recoveries 60%-64% of OOIP) in assays performed using a crude oil with a viscosity of 274 mPa s [11]. In the case of WL gum (an EPS produced by *Sphingomonas* sp. WG) a tertiary recovery of 14.5% was achieved in sand-pack column assays (oil viscosity 300 mPa s), similar to the results obtained with HPAM (13.3%) and xanthan gum (14.8%), resulting in final recoveries between 70% and 75% [5]. Jang and co-workers [10] studied the applicability of xanthan gum in MEOR, in comparison to HPAM. Using a heavy oil (450 mPa s), final recoveries between 67% and 69% were obtained with xanthan gum, whereas in the case of HPAM, they were around 62% [10].

## 4. Conclusions

A low-cost culture medium was developed for EPS production by *R. viscosum* CECT908, using sugarcane molasses (60 g/L) and CSL (10 mL/L) as sole substrates. The amount of EPS produced using the low-cost medium (EPS_CSLM_) was around 6 g/L, almost twice the amount produced using the conventional synthetic medium (EPS_Syn_), which contains glucose and yeast extract. As a result, the cost of the substrates necessary to produce 1 kg of EPS was reduced almost 30 times. Although EPS_CSLM_ and EPS_Syn_ displayed a similar carbohydrate composition, EPS_CSLM_ exhibited a higher viscosifying activity when compared with EPS_Syn_. Consequently, more studies are necessary to understand their different properties. Finally, the applicability of EPS_CSLM_ in MEOR was demonstrated in laboratory sand-pack column assays using a heavy crude oil, where it allowed the recovery of approximately 50% of the entrapped oil, showing a better performance than xanthan gum.

## Figures and Tables

**Table 2:** Sugars composition of polysaccharides produced by *Rhizobium viscosum* CECT908 grown in synthetic (EPS_Syn_) and low-cost medium (EPS_CSLM_), and acetic acid released from them.

Sample	Sugars Composition (mol %)						Total Sugars (mg/g)	Acetic Acid (mg/g)
	Ara	Xyl	Man	Gal	Glc	UA		
EPS_Syn_			3.2	42.4	31.9	22.6	464.4 ± 0.9	213.3 ± 21.3
EPS_CSLM_	1.5	0.7	2.0	43.7	35.0	17.1	567.7 ± 22.7	184.4 ± 7.3

Ara: arabinose; Gal: galactose; Glc: glucose; Man: mannose; UA: uronic acids; Xyl: xylose.

**Table 3:** Glycosidic linkage composition (% mol) of *Rhizobium viscosum* CECT908 EPS.

	% mol			
Glycosyl Linkage	EPS_Syn_	RSD	EPS_CSLM_	RSD
*t*-Ara*f*			2.9	11
3-Ara*f*			0.5	1
5-Ara*f*			0.6	31
Total			3.9	12
*t*-Xyl*p*			0.3	29
4-Xyl*p*			2.3	8
2,4-Xyl*p*			1.2	17
Total			3.8	13
*t*-Man	2.6	5	Traces	
2-Man	1.6	6		
6-Man	1.2	17		
2,6-Man	2.8	8		
Total	8.3	2		
*t*-Gal	0.7	18	0.9	28
4-Gal	50.4	2	50.8	1
2,4-Gal			0.8	24
3,4-Gal			0.6	21
3,6-Gal			0.7	5
4,6-Gal			0.2	25
Total	51.1	2	54.0	0.1
*t*-Glc	2.7	4	2.9	24
4-Glc	14.1	0.2	12.8	4
6-Glc	1.2	2.9	5.0	7
2,4-Glc			0.3	14
3,4-Glc			0.5	5
4,6-Glc	1.5	0.8	1.7	11
2,3,4,6-Glc	2.4	28	1.2	32
Total	21.8	2	24.3	9
*t*-ManA	1.9	16	2.5	27
4-ManA	16.9	8	11.4	33
Total	18.8	6	14.0	16
% Branching	9.0		8.5	

**Table 4:** Economic analysis for the cost of the substrates used for exopolysaccharide production by *Rhizobium viscosum* CECT908.

Substrate	Concentration (g/L Medium)	Price (€/kg) ^a^	Cost of Substrate/L Medium (€)	EPS Titer (g/L)	Cost of Substrate/kg EPS Produced (€)
Glucose	25	0.8	0.02		6.67
Yeast extract	3	72.0	0.216		71.99
K_2_HPO_4_	2	11.3	0.0226		7.53
MgSO_4_·7H_2_O	0.1	5.6	0.00056		0.18
			0.25916	3.0	86.37
SM	60	0.2	0.012		1.97
CSL	13 ^b^	0.4	0.0052		0.85
			0.0172	6.1	2.82

CSL: corn steep liquor. SM: sugarcane molasses. ^a^ The prices were obtained from the corresponding suppliers. ^b^ The density of CSL is 1.3 kg/L.

**Table 5:** Results of sand-pack column assays performed with the exopolysaccharide produced by *Rhizobium viscosum* CECT908 grown in the low-cost medium (EPS_CSLM_) and xanthan gum. The apparent viscosity was measured at 40 °C and the values presented correspond to a shear rate of 9.2 s^−1^. The results represent the average of three independent experiments ± standard deviation.

Treatment	OOIP (mL)	Water Flooding Recovery (% OOIP)	Tertiary Recovery (% OOIP)	Final Recovery (% OOIP)
Water	91.7 ± 2.5	48.4 ± 2.8	-	48.4 ± 2.8
EPS_CSLM_ (50 mPa s)	92.1 ± 2.6	50.7 ± 1.6	25.0 ± 1.1	75.6 ± 2.7
EPS_CSLM_ (100 mPa s)	93.7 ± 2.1	47.7 ± 1.2	26.9 ± 1.2	74.6 ± 0.8
Xanthan (50 mPa s)	94.0 ± 1.7	48.6 ± 1.4	9.2 ± 0.6	57.8 ± 1.7
Xanthan (100 mPa s)	92.2 ± 2.0	50.0 ± 1.1	14.0 ± 1.4	64.0 ± 0.4

## Data Availability

Not applicable.
